# Voluntary childlessness and family planning in men with inflammatory bowel disease: a scoping review

**DOI:** 10.1093/crocol/otag046

**Published:** 2026-06-01

**Authors:** Sara Ma, Victoria Jones, Simona Fourie, Wladyslawa Czuber-Dochan, Paul Galdas

**Affiliations:** School of Health Sciences, York St John University, United Kingdom; School of Health Sciences, York St John University, United Kingdom; Radcliffe Department of Medicine, Oxford University, United Kingdom; Florence Nightingale Faculty of Nursing, Midwifery & Palliative Care, King’s College London, United Kingdom; Department of Health Sciences, University of York, United Kingdom

**Keywords:** inflammatory bowel disease, voluntary childlessness, fatherhood, family planning

## Abstract

**Background:**

Chronic disease can influence family planning decisions, yet this remains under-researched in men. Understanding voluntary childlessness and family planning in men with inflammatory bowel disease (IBD) is essential to inform person-centered, disease-specific, and holistic support. A systematic synthesis of the published literature on childlessness and family planning in men with IBD was required to identify gaps in knowledge and inform future research, policy, and clinical care.

**Methods:**

Scoping review in accordance with the Joanna Briggs Institute guidance and reported in line with the PRISMA-ScR checklist. MEDLINE, EMBASE, CINAHL, and PsychInfo were searched. Two reviewers independently screened and selected relevant studies. Data were extracted, charted and then synthesized using the Patterns, Advances, Gaps, Evidence for practice, and Research recommendations (PAGER) Framework.

**Results:**

A total of 15 papers, reporting data from 1336 men with IBD were included in the review. The findings indicate that IBD may influence men’s reproductive decision-making, with medication safety emerging as a key concern. Men also appear to have poorer reproductive knowledge compared with women with IBD. Few studies have involved partners, and the impact of IBD on partners remains largely undocumented.

**Conclusions:**

This review highlights the significant lack of research, clinical guidance, and patient education regarding men’s family planning decisions in the context of IBD. Men and their partners require tailored, disease-specific information to support informed reproductive choices. Clinicians should recognize the potential impact of IBD on family planning and provide clear guidance on fertility and treatment-related risks, ensuring that management decisions are aligned with patients’ reproductive intentions.

## Graphical Abstract

**Figure:**
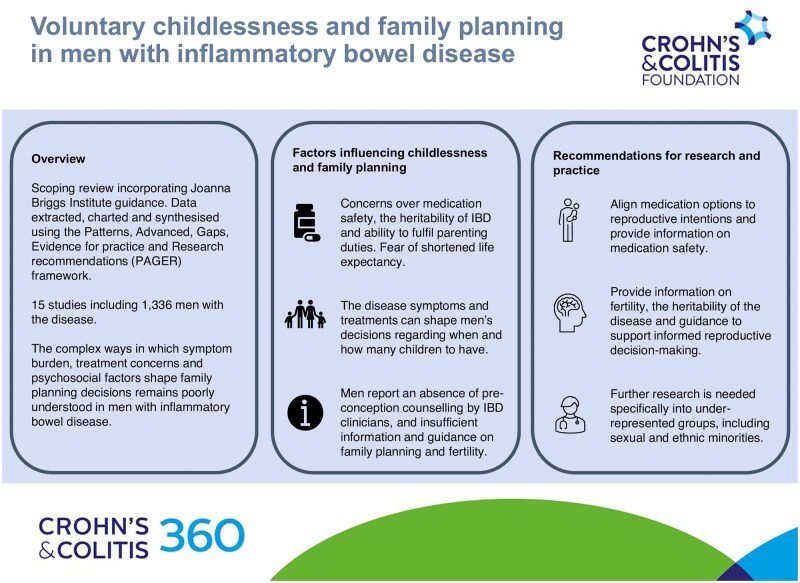

## Introduction

Inflammatory bowel disease (IBD) is a relapsing-remitting, immune-mediated condition of the gastrointestinal (GI) tract. IBD commonly presents with bloody diarrhea, fecal urgency, abdominal pain, malnutrition, and fatigue. It is common for people with the disease to experience extra-intestinal manifestations that affect the joints, eyes, skin, and liver. The pathogenesis of IBD involves a complex interplay of genetic susceptibility, immunopathogenic factors and environmental influences. The 2 most common types of IBD are ulcerative colitis (UC) and Crohn’s disease (CD). There are over 4.9 million people across the world with IBD^1^ and an estimated 744,120 individuals with the disease in the United Kingdom.^2^ Peak incidence occurs between the ages of 17 and 40 years^3^ and therefore affects people when they are likely to be making family planning decisions.

Approximately a quarter of people with IBD report having a family member with the disease.^4^ Overall lifetime risk of developing IBD for first-degree relatives is low (4.8-5.2% in people with CD, 1.6% in people with UC), however the estimated risk for the children with 2 parents with IBD is 33-52%.^5^ Despite the hereditary component of IBD and the impact of the disease symptoms on an individual’s health and overall well-being, family planning decisions in the context of IBD remain poorly understood. A prior review found that 50% of IBD patients lack knowledge about pregnancy-related issues.^6^ One study involving patients with CD demonstrated high demand for information on the hereditary component of IBD.^7^

Childlessness, a term used to describe a person who does not have children, is commonly divided into voluntary and involuntary with the latter term being used to describe medical infertility. This binary division does not fully reflect how fertility and parenthood decisions are multifaceted and involve a combination of physiological and psychosocial considerations. Childlessness can be influenced by age, relationship status, education, financial security, and health.^8^ Considering these so called “situational factors,” it is comprehensible that someone may be involuntarily childless due to reasons outside of medical infertility. While it is recognized that there may be a real-world overlap between voluntary and involuntary childlessness in the context of chronic disease and ill-health, for the purposes of this review involuntary childlessness is used synonymously with medical infertility.

In the United Kingdom, national data sets typically only collect fertility data from women and subsequently there is a lack of data on men.^9^ A similar pattern is evident in IBD research where fertility and childlessness have been more comprehensively examined in women as compared to men.^10^^,^^11^ This imbalance persists despite increasing recognition of the impact of childlessness on men’s identity and health.^12^ Additionally, the focus of research on women reinforces the burden of family-planning and fertility decisions on women.

Within IBD specifically, studies exploring family planning decisions and childlessness have primarily focused on women^10^^,^^13^^,^^14^^,^^15^^,^^16^ leaving the views and needs of men under-represented.^6^ Yet, providing preconception counseling to men and their partners has been identified as an important area of need with the potential to improve perinatal outcomes for both parents.^17^ To deliver such care effectively, clinicians require a deeper understanding of how IBD influences men’s family planning decisions.

### The review

A preliminary search found no reviews on childlessness and parenting decisions in men with IBD. While medical infertility is clearly a relevant issue, the primary focus of this review is voluntary childlessness, particularly the psychosocial aspects of men’s reproductive decision-making in the context of IBD. However, the literature rarely examines voluntary childlessness in isolation. Many studies instead explore broader reproductive decision-making, including fertility knowledge, treatment concerns, and perceptions of parenthood. These factors were therefore considered relevant as they may influence decisions about whether or when to pursue fatherhood. Understanding how men conceptualize fatherhood while managing a chronic condition is crucial for patient-centered care and for addressing gender disparities in reproductive healthcare. The research question guiding this review was: *What is known about voluntary childlessness and the psychosocial aspects of reproductive decision-making in men with IBD?*

### Aims

To systematically identify and synthesize peer-reviewed, published research reporting on family planning decisions and voluntary childlessness in men with IBD. To identify key knowledge gaps and summarize the research methodologies employed in existing studies to inform future research directions.

## Methods

### Design

The scoping review was conducted in accordance with the Joanna-Briggs Institute guidance for scoping reviews^18^ and has been reported according to the Preferred Reporting Items for Systematic reviews and Meta-Analysis, extension for Scoping Reviews (PRISMA-ScR)^19^ (see File S1). A scoping review methodology was chosen for its suitability in mapping the breadth of existing literature and identifying knowledge gaps. Scoping reviews systematically identify and examine relevant research to provide a contextual background to the field of interest, inform priority setting, and unearth suitable methodological approaches for future research.^20^

The Patterns, Advances, Gaps, Evidence for practice, Research recommendations (PAGER) framework^21^ was selected for evidence synthesis and its utility in providing clinically actionable insights relevant to practitioners and researchers.

As this study is a scoping review of published literature and did not involve human participants or primary data collection, ethical approval, and patient consent was not required.

The review protocol was registered in the Open Science Framework and can be found here: https://osf.io/72nx9

### Search methods

The search strategy was developed following consultation with 2 academic librarians across 2 different UK universities (University of York and York St John University). The PCC (Population, Concept and Context) mnemonic was used to guide the search strategy. The population was adult men with a diagnosis of CD or UC, the concept was voluntary childlessness and family planning, and the context was open so that the searches were not restricted by date or geographical region. A combination of controlled vocabulary (MeSH terms) and free terms were used. The full search strategy has been provided in File S2. MEDLINE (via OVID), Embase (via OVID), CINAHL (via EBSCOhost), and PsychInfo (via OVID) were searched. The reference lists of pertinent papers were reviewed alongside hand-searching of key journal websites, institutional library catalogues, and the internet.

### Inclusion and exclusion criteria

Studies reporting data on family planning decisions and childlessness in men with a diagnosis of CD or UC were included. Studies that included men <16 years of age and where data on men was not distinguishable from women were excluded. If the study included participants with other gastrointestinal or inflammatory diseases, the data for CD or UC must have been distinguishable to be included within the review. Primary research studies, including randomized control trials, quasi-experimental, observational, qualitative research, and mixed-method studies were eligible for inclusion. Non-empirical or non-peer reviewed publications (eg, editorials, commentaries, conference abstracts, or proceedings), review articles and papers without primary data were excluded. Only full-text papers published in English were considered. As the focus of this review was voluntary childlessness, studies that did not examine psychosocial aspects of men’s reproductive health or decision-making regarding family planning, such as those solely addressing medical infertility, were excluded. Studies exploring reproductive knowledge, treatment concerns, or perceptions of parenthood were included where these factors may shape reproductive intentions.

### Search outcome

**Figure 1:**
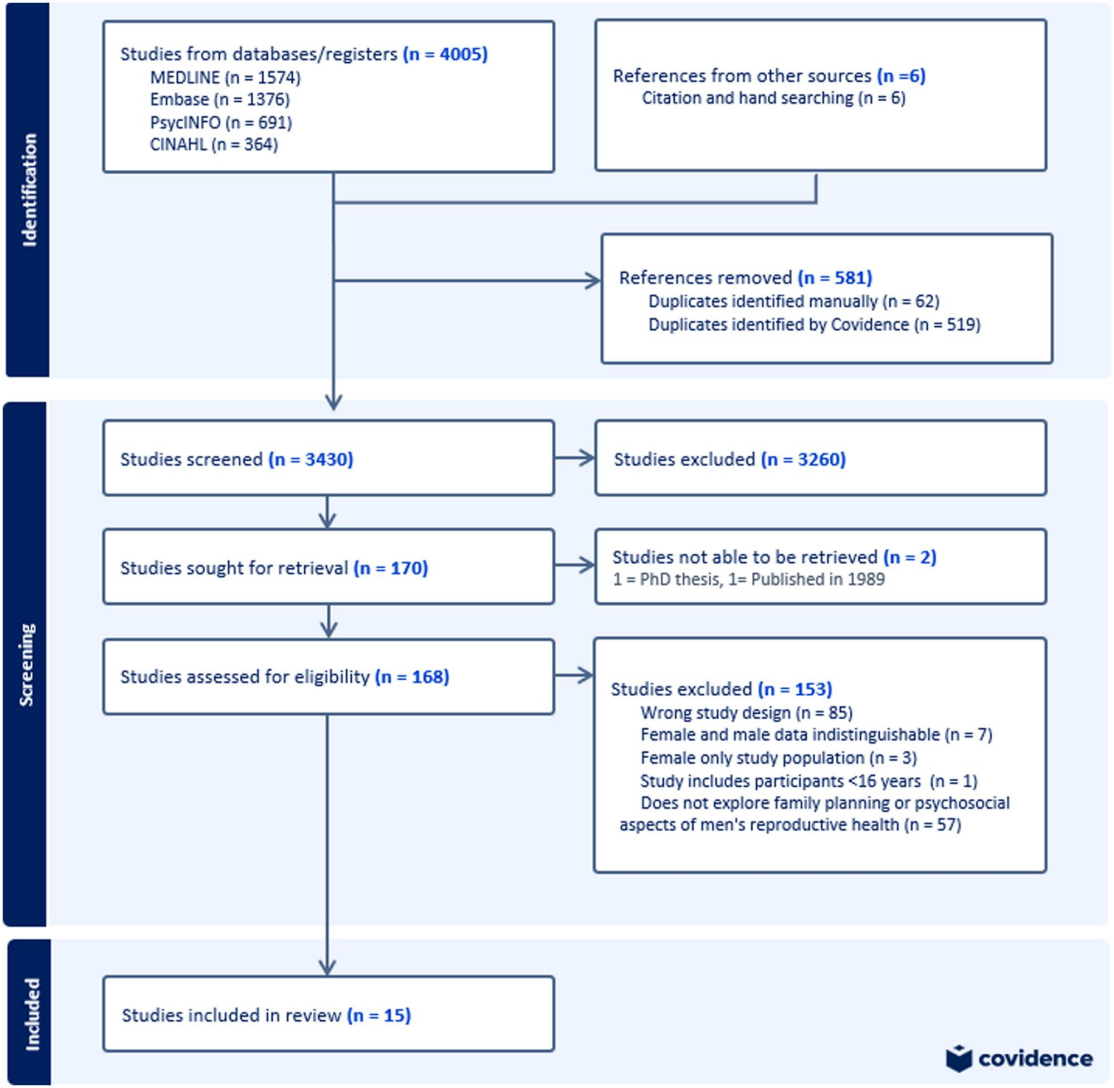
PRISMA flow chart (created by Covidence software and adapted for publication).

The search strategy identified 4011 studies, of which 581 were duplicates (see Figure 1). Covidence, a web-based collaboration software platform was used to support study selection and data extraction. Two reviewers independently screened and selected all records. Titles and abstracts of 3430 studies were screened, with discrepancies resolved through discussion. Full text of 168 studies were then reviewed against the eligibility criteria, and disagreements were resolved by a third reviewer. Ultimately, 15 papers met the inclusion criteria. Of the 5 authors involved in this review, 4 were also members of the authorship teams for 3 of the included studies. The fifth author, who had no prior involvement in any of the included studies, acted as an impartial reviewer. Although, this overlap presented a potential source of bias, the studies were retained due to their relevance and contribution to the field. To ensure objectivity, the impartial reviewer completed the data extraction for these studies.

### Quality appraisal

Consistent with scoping review methodology,^18^ no formal quality appraisal of the studies was conducted.

### Data abstraction

Data was extracted using a piloted form. A single reviewer extracted the data with a second reviewer checking accuracy and correctness of the information. The extraction table included authors, year, country, aim, design, and data collection methods, population, sample size, key findings, and limitations (File S3).

### Synthesis

Data synthesis followed the PAGER framework,^21^ providing a structured and transparent approach. Two reviewers independently examined all 15 included studies and identified key themes inductively. These themes were then charted to enable visual mapping of their distribution across studies. Themes were consolidated into the most pertinent patterns, which were subsequently refined through collaborative discussion. The reviewers developed advances, gaps, implications for practice, policy, and research recommendations aligned with each pattern. This process was iterative and required repeatedly revisiting and reexamining the studies to ensure rigor and coherence. Additional tables and graphs were produced to present the findings clearly and accessibly.

## Findings

The fifteen included studies were published between 1998 and 2024 and were conducted in North America (*n* = 4; Canada, USA), Europe (*n* = 8; UK, Czech Republic, Netherlands, Germany and one multi-country European study), Asia (*n* = 1; Japan), and Australia (*n* = 2). Study designs comprised cross-sectional surveys (*n* = 7, including one secondary analysis), qualitative studies (*n* = 6), one cohort study and one non-randomized experimental study. A full summary of the included studies is presented in Table 1.

**Table 1:** Summary of included studies (in chronological order).

Authors and year	Country	Study aim	Study design	Population characteristics	Total sample size (no. of men)
**Maunder et al.** ^36^	Canada	To identify the concerns and sex differences of people with IBD.	Secondary analysis of cross-sectional survey data	IBD patients ≥18 years	343 (149)
**Mountifield et al.** ^27^	Australia	To determine whether issues surrounding IBD, pregnancy and childbearing influence reproductive behavior.	Cross-sectional postal survey	IBD patients 18-59 years	217 (74)
**Sato et al.** ^25^	Japan	To investigate male opinions about pregnancy, conception and neonatal outcomes for partners.	Cross-sectional survey	IBD patients 20-59 years	364 (364)
**Zelinkova et al.** ^34^	The Netherlands	To assess changes in medication in the peri-conceptional period.	Prospective cohort study	IBD patients with active disease and desire to reproduce 20-52 years	61 (10)
**Mountifield et al.** ^35^	Australia	To examine the effect of a single group education session on IBD-specific reproductive knowledge in subjects with IBD.	Non-randomized experimental study	IBD patients Mean age 40.3 years	155 (48)
**Keller et al.** ^33^	United States	To understand how individuals taking IBD medications during key reproductive periods make decisions about their medication use.	Qualitative research: social media content analysis	IBD patients and family/caregivers Age not reported	1818 (male unknown)
**Duricova et al.** ^23^	Czech Republic	To investigate reproductive behavior in patients with IBD in the Czech Republic.	Cross-sectional survey	IBD patients 18-65 years	792 (272)
**Rao et al.** ^26^	United States	To assess counseling and knowledge about IBD and reproductive health.	Cross-sectional survey	IBD patients 18-45 years	100 (46)
**Fourie et al.** ^28^	UK	To explore patient experiences of intimacy and sexuality in those living with IBD.	Qualitative research: interviews and anonymous narratives via online forms.	People with IBD 17-64 years	43 (11)
**Winter et al.** ^22^	United States	To assess the extent of and risk factors for voluntary childlessness in IBD.	Cross-sectional survey	IBD patients ≥18 years	464 (140)
**Thapwong et al.** ^31^	UK	To explore the lived experience of IBD patients and their family members regarding impacts of IBD on family members and their coping strategies.	Qualitative research: participant interviews	People diagnosed with IBD for at least 1 year plus 1st degree, co-habiting family member 23-61 years	6 participants with IBD (4) 6 partners of participants (2)
**Vieujean et al.** ^24^	Multiple European countries	To investigate healthcare professional and patient knowledge on fertility, pregnancy, and sexual function.	Cross-sectional online survey	Healthcare professionals and adult IBD patients. 27-83 years	793 patients (176)
**Erdmann et al.** ^29^	Germany	To understand how body experiences affect patients with IBD.	Qualitative research: participant interviews	IBD patients 19-44 years	10 (4)
**Gabova et al.** ^32^	Czech Republic	To investigate the perceived impact of IBD on sexual life and family planning.	Qualitative research: participant interviews	People with IBD 21-67 years	36 (16)
**Ma et al.** ^30^	UK	To describe and interpret the sexual health experiences of men with IBD.	Qualitative research: participant interviews	IBD patients 20-66 years.	22 (22)

**Figure 2:**
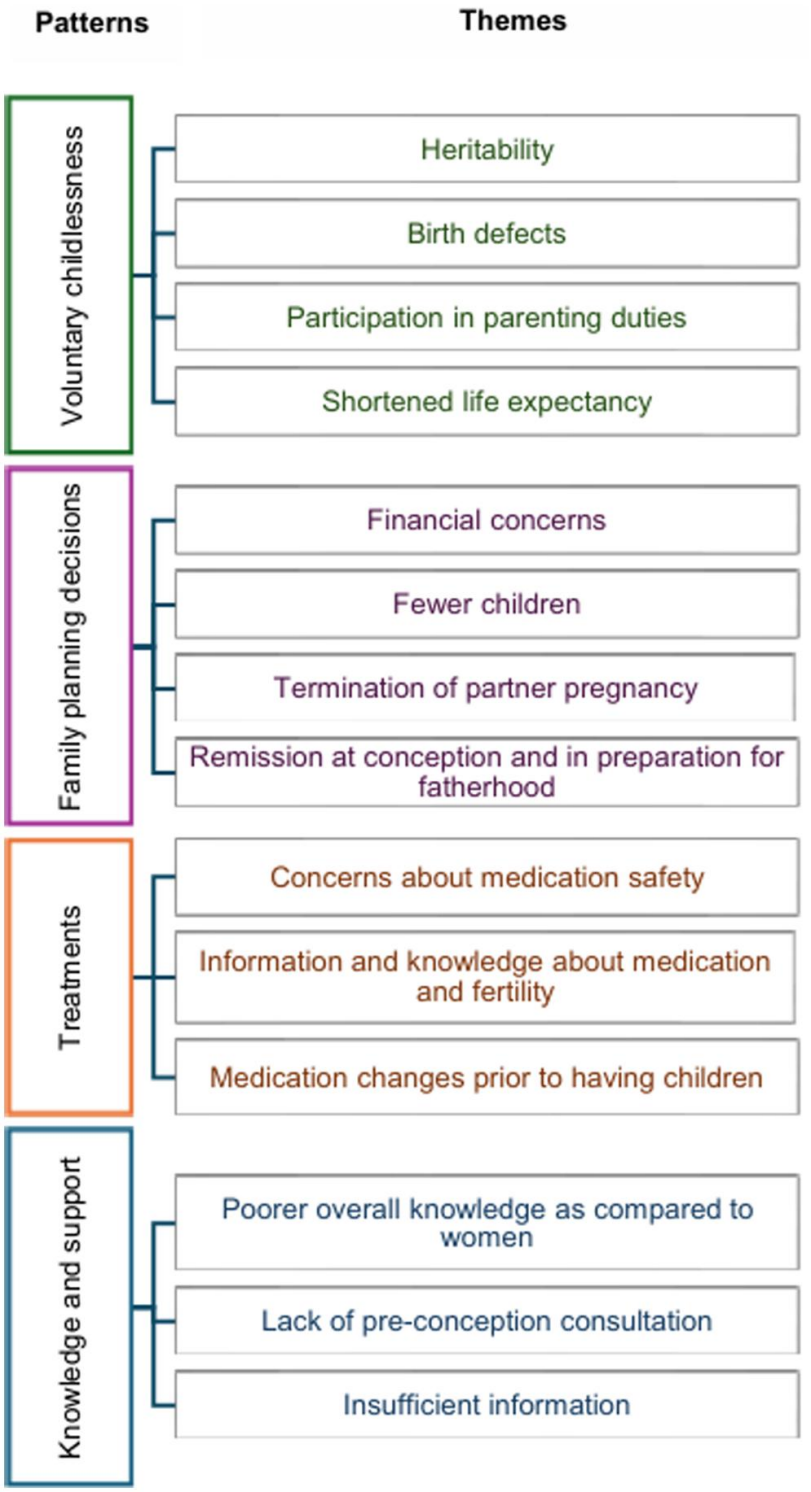
Themes and sub-themes identified across the studies.

Across these studies, 1336 men with IBD were represented, however research specifically focused on men remains sparse. Despite the limited volume of published, peer-reviewed research in this area, several informative themes were identified. Figure 2 and Table 2 present the themes and overarching patterns: (1) voluntary childlessness; (2) family planning decisions; (3) IBD treatments; and (4) knowledge and support.

**Table 2:** Findings organized according to the PAGER framework (Bradbury-Jones et al. 2022).

Pattern	Advances	Gaps	Evidence for practice	Research recommendations
**Understanding voluntary childlessness**	Men report concerns over heritability, shortened life expectancy, risk of birth defects, and ability to care for children.	Rates of voluntary childlessness in men with IBD. How IBD affects reproductive decisions in men.	Acknowledge IBD’s possible role in reproductive choices. Facilitate open, non-judgmental discussions about reproductive intentions and concerns.	Establish the prevalence of voluntary childlessness in men with IBD. Explore the disease-specific influences on men’s reproductive intentions.
**Family planning decisions**	The disease may alter when and how many children men decide to have.	How IBD influences family planning decisions in men. Partner perceptions toward family planning.	Assess and revisit family planning intentions throughout disease and life course. Align treatment decisions with reproductive goals.	Examine the barriers that IBD may pose to fatherhood and what support may be acceptable and valuable. Investigate partner and family perspectives and needs.
**IBD treatments**	Medication safety is a key concern. Men may alter or delay treatment or deprioritize family planning due to disease management plans.	How IBD treatments influence reproductive intentions. How reproductive goals might affect treatment preferences and adherence.	Provide information on the risks and benefits of medication to fertility. Misconceptions regarding medication safety should be identified and addressed.	Clarify treatment-related concerns and how these might affect reproductive intentions. Ascertain how treatment related concerns can be better supported by healthcare practice.
**Knowledge and support**	Men have lower reproductive knowledge as compared to women with IBD and report that they do not receive family planning information or consultation.	What is the current state of service provision in regard to reproductive care for men with IBD. What information and healthcare provision is required by men with IBD.	Incorporate family planning discussions into routine care. Offer written and verbal education that includes the perspective and needs of men and their partners. Provide multidisciplinary input that considers physiological, and psychosocial well-being in relation to reproductive planning.	Determine the informational needs of men and the most acceptable and accessible ways to deliver these. Survey the current provision of fertility and family planning information and healthcare provision for men with IBD. Evaluate the outcomes of pre-conception counseling and information in male IBD populations.

### Understanding voluntary childlessness

Reported rates of voluntary childlessness among men with IBD varied widely; 7.8%,^22^ 18.1%,^23^ and 27.3%.^24^ Differences in recruitment strategies and data collections methods mean these findings should not be directly compared. Furthermore, as none of these studies included control groups, it is not possible to determine whether voluntary childlessness is more common in men with IBD as compared to the general population or other disease and disability states.

Sato et al. reported that 24.4% of men (*n* = 71) were hesitant to have children due to IBD, with significantly higher rates in men with CD as compared with those with UC.^25^ In the study by Ďuricová et al., 42.3% of voluntarily childless men cited IBD as the primary reason.^23^ Among these men, 50% expressed concern about the genetic risk of IBD, 34.5% feared they would be unable to care for a family, and 15.4% were worried about medication safety. Similarly, Rao et al. found that 15% of men had considered not having children due to IBD, with concerns focused on heritability (86%), caregiving ability (43%), and medication effects (29%).^26^

Sociodemographic factors also appeared to influence childlessness. Winter et al. reported that voluntarily childless participants were younger, had lower levels of formal education and income, and were more likely to be permanently disabled than those who wished to have children.^22^ Although voluntary childless participants were diagnosed at a younger age and had a longer disease duration, IBD type, hospitalizations, and surgeries were not associated with an increased likelihood of voluntary childlessness. However, as the data were not disaggregated by sex, interpretation is limited. Similarly, Mountifield et al. identified fear of IBD-related congenital abnormalities, genetic risk, medication effects, medical advice against conception, and fatigue as reasons for voluntary childlessness but did not specify whether these responses were provided by men, women or both.^27^

Qualitative studies provided further insights. Participants described concerns about medication effects,^28^ fear of shortened life expectancy,^29^ and the need to prioritize their own health over parenting responsibilities.^30^ One partner of a man with IBD also expressed concern over the hereditary nature of CD.^31^

In summary, although several studies have reported rates of voluntary childlessness among men with IBD, population-level data are lacking and comparisons with the general male population are not possible. Existing evidence suggests that voluntary childlessness may be driven by disease-related concerns, particularly fear about heritability, reduced life expectancy and the ability to fulfill a parental role while managing a chronic illness.

### Family planning decisions

In addition to influencing decisions about whether to become a father, the included papers suggest that IBD may also shape men’s family planning decisions. In the study by Ďuricová et al. only 11% of men (*n* = 30) reported that IBD had affected their reproductive plans; of these, one-third (33.3%) stated they had decided to have fewer children than initially intended.^23^ In contrast, Winter et al. reported that 93.4% of men (*n* = 85) who had, or wanted to have children, planned for fewer children because of IBD.^22^ Similarly, one participant in the Thapwong et al. study described financial and lifestyle pressures, alongside disease related concerns, that led him and his partner to limit family size to one child.^31^ Another participant in the Erdmann et al. described mixed feelings, fear as well as joy, about having a third child.^29^ However, across both qualitative studies it remained unclear to what extent IBD itself influenced the decisions about family size.^29^^,^^31^

Ma et al. reported that men perceived IBD as a barrier to parenting, influencing decisions about the timing of children: *“I need to be healthy to be able to look after a child or two and already I’m at a disadvantage because of fatigue, because of pain, because of illness”*.^30^ Mountifield et al. examined pregnancy termination rates among women with IBD and the female partners of male IBD patients (16.5% in CD vs 15.3% in UC) with 17.7% of terminations attributed to IBD.^27^ However, the findings were not disaggregated by gender, and no other studies explored how IBD may influence decisions regarding termination of pregnancy. Ďuricová et al. also reported that 30.5% of men had fathered children unintentionally,^23^ though contraceptive choices were not discussed across the included studies. The perspectives of partners were similarly absent. In one qualitative study, men did not raise concerns regarding family planning, whereas women did.^32^ However, men did describe concerns regarding sexual activity and relationships, both of which may indirectly influence reproductive decisions.

Sato et al. reported that 41.4% of men (*n* = 151) believed disease remission was necessary at the time of conception, and 19.2% (*n* = 70) considered a medication-free state essential.^25^ Finally, one participant in the Ma et al. study, a man in a same-sex relationship, described having considered adoption but deciding against it following multiple surgeries.^30^ This single account highlights the broader lack of representation of sexual minority groups in the existing literature.

### IBD treatments

Several studies highlighted the influence of IBD treatments on men’s reproductive intentions. Concerns around medication safety were prominent. Sato et al. reported that more than half of male participants (51.4%, *n* = 187) considered the safety of medication used to induce and maintain remission to be most important issue when planning to conceive.^25^ Uncertainty was also evident, with 29.4% (*n* = 82) of men unsure about the safety of their medication in relation to conception.^23^ Perceived risks of treatment-related infertility were also a concern. Mountfield et al. described a case in which a man taking sulfasalazine had attempted to conceive for 15 years before becoming aware of the drug’s reversible infertility effects.^27^ Similarly, posts from online patient forums revealed confusion and anxiety regarding male infertility and potential birth defect risks linked to IBD medications, with some individuals expressing surprise that such effects were possible.^33^ Treatment modification in the context of family planning were also reported. Zelinkova et al. found that men changed or discontinued therapy following advice from their gastroenterologist, including delaying or stopping medications such as methotrexate, anti-TNF agents and 6-mercaptopurine.^34^ Surgical treatment also shaped reproductive decision-making. In one qualitative study, a participant described reluctance to undergo proctectomy due to fears of impotence and impacts on fertility, while another reported delaying stoma reversal to maintain stability in his health and family life.^30^

Collectively, these findings indicate that medication effects and surgical interventions are important considerations for men with IBD when planning parenthood. They also highlight substantial gaps in understanding and awareness, which are explored further in the next section.

### Knowledge and support

The included papers suggest that men with IBD have lower levels of reproductive and fertility-related knowledge than women. Mountifield et al. reported significantly lower baseline CCPKnow scores in men, with 85.4% of men (*n* = 41) demonstrating poor knowledge compared to 56.1% of women (*n* = 60).^35^ Rao et al. similarly found low median CCPKnow scores in men with only 17% achieving an adequate score.^26^ Although infertility concerns were reported more frequently among women, a substantial minority of men (32.5%) also expressed worry about their fertility.^27^

Despite these knowledge gaps, family planning information and counseling were not routinely provided. Rao et al. found only 30% of men had received counseling about heritability, fertility or medication use.^26^ Ďuricová et al. similarly reported that just 20.2% of men (*n* = 55) had ever discussed reproductive issues with a clinician.^23^ Although satisfaction with the advice received was high (76.4%), Vieujean et al. observed that nearly three-quarters of men (73.9%) had not received pre-conception counseling (vs 62.1% of women) and over half of men (52.2%) felt they lacked sufficient information about IBD and pregnancy.^24^ Most men nonetheless reported feeling comfortable discussing these topics with their IBD specialist (75.4%).^24^

Little evidence exists to guide the format of support preferred by men. Rao et al. found that participants preferred written material (84%) and clinic-based discussions (69%),^26^ although these findings were derived from a mixed-gender sample and therefore cannot be interpreted as reflecting the preferences of men specifically. Winter et al. reported greater preference for face-to-face discussions over online resources among their mixed-gender sample.^22^ Winter et al. also noted that most participants (both men and women) did not believe pre-conception counseling would alter decision regarding having children, but it may reduce anxiety.^22^ Maunder et al. found that when ranking 25 IBD-related concerns, having children was rated lowest by men.^36^ It is unclear whether this ranking reflects low concern about fertility or the broader considerations regarding parenthood, however, energy levels, medication effects, and disease unpredictability were the most highly ranked, all of which may indirectly influence family planning. As several studies did not report sex-disaggregated findings, conclusions regarding men’s specific informational preferences remain tentative.

Overall, while reproductive health may not be a priority concern for many men with IBD, the studies reviewed demonstrate that concerns about fertility, medication safety, and heritability do exist. These concerns coexist with clear gaps in knowledge and limited clinician-led counseling, highlighting a need for tailored, accessible, and acceptable education and support.

## Discussion

This original review comprehensively and systematically identified and synthesized the published literature on voluntary childlessness in men with IBD. It was found that voluntary childlessness and family planning in men with IBD remains poorly understood and under-researched. Many included studies explored reproductive decision-making, fertility knowledge, or treatment concerns rather than voluntary childlessness explicitly; however, these factors represent key influences on men’s decisions about whether or when to pursue fatherhood. Only 15 studies met the inclusion criteria, of which just 2 focused exclusively on men. Most were conducted in high-income countries and lacked representation of ethnically diverse populations or sexual minority groups, limiting generalizability. Population-level studies suggest differences in parenthood rates among people with IBD, although distinctions between voluntary and involuntary childlessness are rarely made. Furthermore, many studies in the existing literature examine reproductive intentions, fertility knowledge, or treatment-related concerns rather than voluntary childlessness explicitly. While these topics do not directly measure voluntary childlessness, they represent important influences on reproductive decision-making and may shape decisions regarding whether to pursue parenthood. A German cohort study reported higher rates of childlessness in both men and women with IBD as compared to controlled.^37^ A large Swedish registry study of over 29,104 men found reduced birth rates among men with IBD compared with matched controls (1.28 vs 1.35 per 1000-person-years, *P* < 0.001).^38^ A systematic review likewise reported fewer children among men with CD but not UC.^39^ However, none of these studies examined underlying motivations or psychosocial influences.

Across the reviewed literature, disease-specific factors such as heritability concerns, medication effects and perceived ability to parent while living with a chronic illness appear to influence decision-making for some men. Qualitative studies also describe emotional and practical challenges, including fatigue, uncertainty about long-term health and financial constraints.^28^^,^^29^^,^^30^^,^^31^ However, several qualitative studies included very small numbers of male participants, which may limit the extent to which the findings represent men’s experiences, particularly across varying socioeconomic and ethnic contexts. These findings should therefore be interpreted as preliminary insights rather than definitive evidence of all men’s experiences. Although, having children ranked low amongst men’s IBD-related concerns,^35^ this may reflect broader life priorities rather than an absence of concerns about fertility or medication safety.^23^^,^^25^^,^^26^

Treatment safety was a central theme. Concerns about medication effects on fertility were widespread, with more than half of men in one study identifying medication safety as the most important issue when planning conception.^25^ Some medications carry known risks, eg, sulfasalazine can cause reversible oligospermia^40^ and methotrexate has historically been avoided, although emerging evidence suggests minimal impact on male fertility.^41^ Other commonly used therapies, such as anti-integrins, anti-IL 12/23 inhibitors, and calcineurin inhibitors, have not been shown to impair sperm quality.^42^ The reviewed studies largely pre-date recent European guidance on fertility in IBD,^42^ which may explain reports of men being advised to stop or modify treatment before attempting conception.^34^

Surgical interventions also influenced reproductive decisions. One man delayed proctectomy due to concerns about erectile dysfunction and fertility^30^^,^^43^; another postponed stoma reversal to maintain stability for his family.^30^ While men in the Sato et al. study felt that remission was required prior to conception,^25^ there are no known risks to maternal and fetal health if conception occurs when the father has active disease, however, active disease may affect fertility.^42^ Evidence regarding the impact of surgery on male fertility is mixed: some operations may increase the risk of erectile dysfunction,^43^ whereas improved disease control after ileal pouch–anal anastomosis may enhance fertility despite potential sexual side effects.^44^ Given rapid developments in IBD management, clinicians must remain up-to-date on treatment safety and effectively communicate risks and options to patients.

Men’s reproductive knowledge was consistently low across studies. Although CCPKnow is the only validated tool available, it was developed for women and does not fully reflect men’s information needs.^45^ However, CCPKnow was originally developed and validated for women, and some items focus on pregnancy-related issues that may be less directly relevant to men. Lower scores among men may therefore partly reflect limitations of the measurement instrument rather than solely a true deficit in reproductive knowledge.

**Figure 3:**
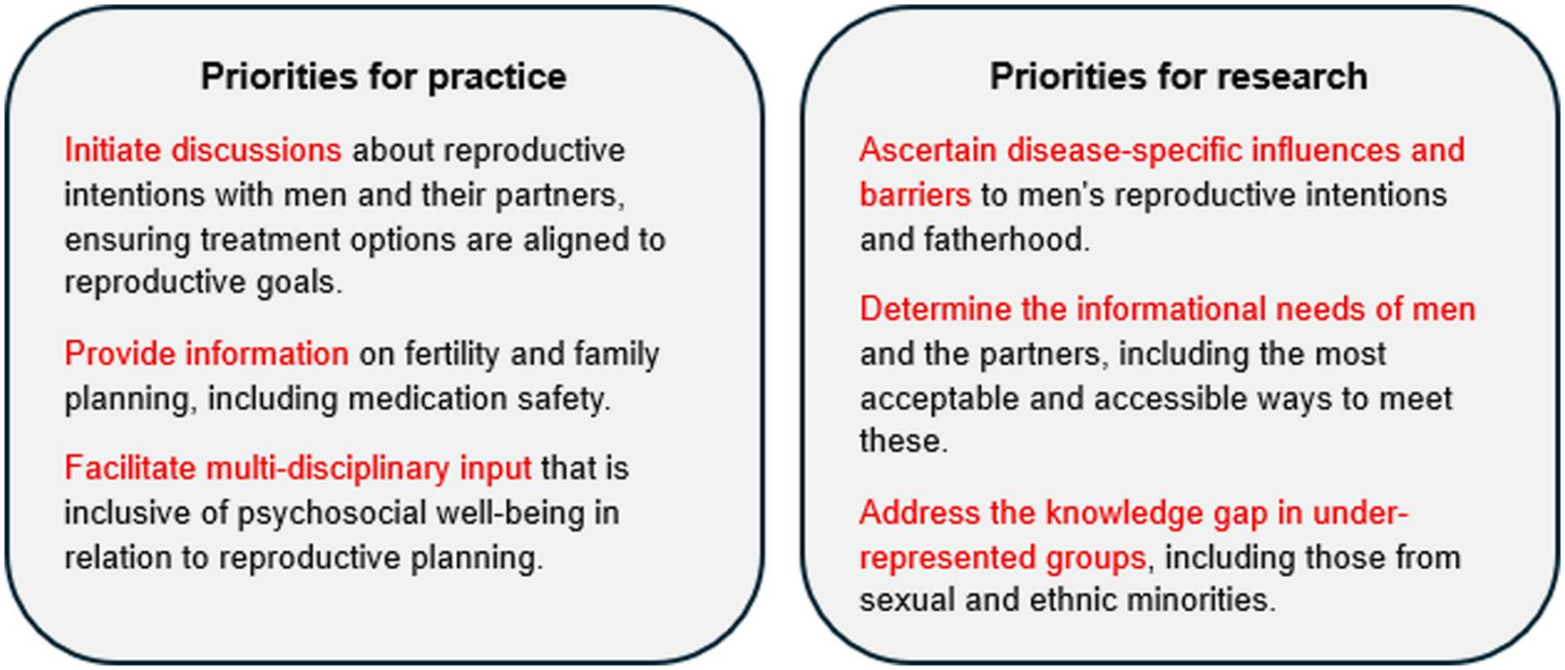
Priorities for practice and research.

A preference for face-to-face consultation was unearthed in one paper within this review,^22^ although reasons for this were not given. The patient-physician relationship has been identified as a key driver in treatment decision making in IBD^46^ and patients value discussions that address or clarify information they have accessed independently.^47^ It has been reported that IBD consultations often prioritize disease activity over broader QoL issues, leaving limited opportunity to discuss reproductive intentions.^48^ Understanding men’s reproductive goals is important not only for treatment planning but also for holistic care. Reproductive health is linked to broader well-being and fatherhood has been associated with reduced suicide risk.^49^ Wider population studies suggest associations between childlessness and adverse health outcomes, including cardiovascular risk.^50^ Whether these patterns apply to men with IBD is unknown. Major evidence gaps also remain regarding partners’ perspectives and the experiences of men from ethnically diverse backgrounds and sexual minority groups. These gaps highlight clear priorities for clinical practice and future research, summarized in Figure 3.

### Limitations

To our knowledge, this is the first literature review to focus specifically on voluntary childlessness and reproductive decision-making in men with IBD. The review adopted a systematic and transparent approach to study selection and data extraction. The use of the PAGER framework enabled a structured and conceptually grounded synthesis of findings. All searching, screening, and data extraction were conducted independently by 2 reviewers, enhancing methodological rigor and reducing the risk of bias. By integrating qualitative and quantitative evidence, the review offers a comprehensive overview of an understudied area and identifies clear gaps and priorities for future research and clinical practice.

The review is subject to several limitations, many of which stem from constraints within the available evidence base. There is a paucity of research on voluntary childlessness and family planning in men with IBD, with only 2 studies^25^^,^^30^ focusing exclusively on male participants. Several included studies examined broader reproductive decision-making or fertility concerns rather than voluntary childlessness explicitly, which limits the ability to clearly distinguish voluntary childlessness from infertility-related childlessness within the available literature. Additionally, the primary instrument used to assess reproductive knowledge (CCPKnow) was developed for women and may not fully capture men’s knowledge needs or priorities. As summarized in Table 1, most studies were cross-sectional or qualitative with small sample sizes and varying definitions of voluntary childlessness. Additionally, studies were mostly conducted within single-center settings, which limit generalizability and prevents robust estimation of the prevalence of voluntary childlessness. Many studies also lacked sex-disaggregated analyses or comparator populations, further limiting interpretation of gender-specific findings and preventing comparisons with the general population. Reported prevalence should therefore be interpreted cautiously. Demographic data were frequently incomplete, particularly regarding ethnicity, socioeconomic status and sexual orientation, and gay or bisexual men were almost entirely absent from the literature, raising concerns about representativeness. A likely selection bias toward participants from high-income and high-resource settings is also evident, which is important considering that family planning decisions are shaped by cultural and societal contexts.

Methodological limitations were also common. Few studies included control groups, making it difficult to determine whether rates or drivers of voluntary childlessness differ from those in the general population. Sex-disaggregated data were inconsistently reported, preventing clear interpretation of gender-specific findings. In several studies, some findings were reported from mixed-gender samples without separate analysis for men, meaning that certain conclusions regarding informational needs and reproductive concerns should be interpreted cautiously. Furthermore, the available evidence does not permit exploration of how age, IBD phenotype, treatment type, or disease activity influence men’s reproductive intentions or decision-making. Taken together, the evidence base is sparse and heterogeneous, meaning that conclusions should be interpreted with caution.

Finally, several members of the review team were authors on 3 of the included studies. To minimize potential influence of this on the findings of the review, an independent reviewer with no prior involvement in these studies conducted the data extraction for these papers; however, some residual risk of unintentional influence cannot be completely excluded.

## Conclusion

This scoping review synthesized the limited but emerging evidence on voluntary childlessness and family planning among men with IBD. While fatherhood may not be a primary concern for all men, a significant proportion express concerns about fertility, heritability, treatment safety and their ability to parent while managing a chronic illness. Medication and surgical treatments play a notable role in shaping reproductive decision-making, yet many men report inadequate access to tailored information or pre-conception counseling. Representation of ethnically diverse and sexual minority groups is largely absent, and the perspectives of partners remain under-explored.

Addressing these gaps through inclusive, methodologically robust research is a priority. However, clinicians need not wait for further evidence to initiate discussions with men about their reproductive intentions. Proactive, person-centered conversations, supported by accurate, accessible information, are essential to ensure that decisions align with men’s values, health needs and life goals. Finally, reproductive decision-making should be understood within the broader context of psychosocial wellbeing, which is best supported through multidisciplinary care in both practice and research.

## Supplementary Material

otag046_Supplementary_Data

## Data Availability

This review is based on previously published data. No new datasets were generated or analyzed during this review, and data sharing is not applicable.

## References

[1] Wang R , Li Z, Liu S, Zhang D. Global, regional and national burden of inflammatory bowel disease in 204 countries and territories from 1990 to 2019: a systematic analysis based on the global burden of disease study 2019. BMJ Open. 2023;13:e065186. 10.1136/bmjopen-2022-065186 PMC10069527 36977543

[2] King D , Reulen RC, Thomas T, et al Changing patterns in the epidemiology and outcomes of inflammatory bowel disease in the United Kingdom: 2000–2018. Aliment Pharmacol Ther. 2020;51:922-934. 10.1111/apt.15701 32237083

[3] Pasvol TJ , Horsfall L, Bloom S, et al Incidence and prevalence of inflammatory bowel disease in UK primary care: a population-based cohort study. BMJ Open. 2020;10:e036584. 10.1136/bmjopen-2019-036584 PMC7371214 32690524

[4] Cleynen I , Boucher G, Jostins L, et al International Inflammatory Bowel Disease Genetics Consortium. Inherited determinants of Crohn’s disease and ulcerative colitis phenotypes: a genetic association study. Lancet. 2016;387:156-167. 10.1016/S0140-6736(15)00465-1 26490195 PMC4714968

[5] Ek WE , D’Amato M, Halfvarson J. The history of genetics in inflammatory bowel disease. Ann Gastroenterol. 2014;27:294-303. https://pmc.ncbi.nlm.nih.gov/articles/PMC4188925/. 25331623 PMC4188925

[6] Purewal S , Chapman S, Czuber‐Dochan W, Selinger C, Steed H, Brookes MJ. Systematic review: the consequences of psychosocial effects of inflammatory bowel disease on patients’ reproductive health. Aliment Pharmacol Ther. 2018;48:1202-1212. 10.1111/apt.15019 30411389 PMC6587548

[7] Wu Q , Zhong J. Disease-related information requirements in patients with Crohn’s disease. Patient Prefer Adherence. 2018;12:1579-1586. 10.2147/PPA.S169706 30214160 PMC6118336

[8] Gray E , Evans A, Reimondos A. Childbearing desires of childless men and women: When are goals adjusted? Adv Life Course Res. 2013;18:141-149. 10.1016/j.alcr.2012.09.003 24796265

[9] Berrington A. Childlessness in the UK. In: Kreyenfeld M, Konietzka D, eds. Childlessness in Europe: Contexts, Causes, and Consequences. Springer; 2017:57-76. 10.1007/978-3-319-44667-7_3

[10] Sulz MC , Doulberis M, Fournier N, et al Swiss IBD Cohort Study Group. Childlessness in patients with inflammatory bowel disease: data from the prospective multi-center Swiss IBD cohort study. J Gastrointestin Liver Dis. 2023;32:460-468. 10.15403/jgld-5132 38147613

[11] Palomba S , Sereni G, Falbo A, et al Inflammatory bowel diseases and human reproduction: a comprehensive evidence-based review. World J Gastroenterol. 2014;20:7123-7136. 10.3748/wjg.v20.i23.7123 24966584 PMC4064059

[12] Hadley RA. Muted voices of invisible men: the impact of male childlessness. In: Wilkinson K, Woolnough H, eds. Work-Life Inclusion: Broadening Perspectives across the Life-Course. Emerald Publishing Limited; 2024: 135-146. 10.1108/978-1-80382-219-820241011

[13] Selinger CP , Ghorayeb J, Madill A. What factors might drive voluntary childlessness in women with IBD? Does IBD-specific pregnancy-related knowledge matter? J Crohns Colitis. 2016;10:1151-1158. 10.1093/ecco-jcc/jjw078 26989194

[14] Gawron LM , Goldberger AR, Gawron AJ, Hammond C, Keefer L. Disease-related pregnancy concerns and reproductive planning in women with inflammatory bowel diseases. J Fam Plann Reprod Health Care. 2015;41:272-277. 10.1136/jfprhc-2014-101000 25902816 PMC8451967

[15] Huang VW , Chang HJ, Kroeker KI, et al Does the level of reproductive knowledge specific to inflammatory bowel disease predict childlessness among women with inflammatory bowel disease? Can J Gastroenterol Hepatol. 2015;29:95-103. 10.1155/2015/715354 25803020 PMC4373568

[16] Toomey D , Waldron B. Family planning and inflammatory bowel disease: the patient and the practitioner. Fam Pract. 2013;30:64-68. 10.1093/fampra/cms035 22843639

[17] Kothari A , Thayalan K, Dulhunty J, Callaway L. The forgotten father in obstetric medicine. Obstet Med. 2019;12:57-65. 10.1177/1753495X18823479 31217809 PMC6560841

[18] Peters MD , Marnie C, Tricco AC, et al Updated methodological guidance for the conduct of scoping reviews. JBI Evid Synth. 2020;18:2119-2126. 10.11124/JBIES-20-00167 33038124

[19] Tricco AC , Lillie E, Zarin W, et al PRISMA extension for scoping reviews (PRISMA-ScR): checklist and explanation. Ann Intern Med. 2018;169:467-473. 10.7326/M18-0850 30178033

[20] Pollock D , Davies EL, Peters MD, et al Undertaking a scoping review: a practical guide for nursing and midwifery students, clinicians, researchers, and academics. J Adv Nurs. 2021;77:2102-2113. 10.1111/jan.14743 33543511 PMC8049063

[21] Bradbury-Jones C , Aveyard H, Herber OR, Isham L, Taylor J, O’Malley L. Scoping reviews: the PAGER framework for improving the quality of reporting. Int J Soc Res Methodol. 2022;25:457-470. 10.1080/13645579.2021.1899596

[22] Winter RW , Boyd T, Chan WW, Levy AN, Friedman S. Risk factors for voluntary childlessness in men and women with inflammatory bowel disease. Inflamm Bowel Dis. 2022;28:1927-1931. 10.1093/ibd/izac104 35640111

[23] Ďuricová D , Kratka Z, Bortlik M, et al Inflammatory bowel disease had a negative impact on patients’ reproductive behaviour: the first multicentre survey in the Czech Republic. Gastroenterol Hepatol. 2021;75:12-19. 10.48095/ccgh202112

[24] Vieujean S , De Vos M, D’Amico F, et al Inflammatory bowel disease meets fertility: a physician and patient survey. Dig Liver Dis. 2023;55:888-898. 10.1016/j.dld.2023.01.149 36697343

[25] Sato A , Naganuma M, Asakura K, et al Conception outcomes and opinions about pregnancy for men with inflammatory bowel disease. J Crohns Colitis. 2010;4:183-188. 10.1016/j.crohns.2009.10.004 21122503

[26] Rao AK , Zikos TA, Garay G, Lee KE, Streett SE. Patients report infrequent counseling by physicians and inadequate knowledge about inflammatory bowel disease and reproductive health issues. Am J Perinatol. 2023;40:1651-1658. 10.1055/s-0041-1740193 34902866

[27] Mountifield R , Bampton P, Prosser R, Muller K, Andrews JM. Fear and fertility in inflammatory bowel disease: a mismatch of perception and reality affects family planning decisions. Inflamm Bowel Dis. 2009;15:720-725. 10.1002/ibd.20839 19067431

[28] Fourie S , Norton C, Jackson D, Czuber‐Dochan W. Grieving multiple losses: Experiences of intimacy and sexuality of people living with inflammatory bowel disease: a phenomenological study. J Adv Nurs. 2024;80:1030-1042. 10.1111/jan.15879 37788088

[29] Erdmann A , Rehmann-Sutter C, Schrinner F, Bozzaro C. The body as an obstacle and the “other”: how patients with chronic inflammatory bowel diseases view their body, self and the good life. BMC Med Ethics. 2024;25:82. 10.1186/s12910-024-01076-2 39049028 PMC11267929

[30] Ma S , Knapp P, Galdas P. “My sexual desires, everything, my normal life just stops”: a qualitative study of male sexual health in inflammatory bowel disease. J Clin Nurs. 2024;33:4034-4047. 10.1111/jocn.17292 38797921

[31] Thapwong P , Norton C, Terry H, Czuber-Dochan W. Impact of inflammatory bowel disease on partners: a qualitative study. Gastrointestinal Nursing. 2022;20:40-50. 10.12968/gasn.2022.20.3.40

[32] Gabova K , Bednarikova H, Meier Z, Tavel P. Exploring intimacy and family planning in inflammatory bowel diseases: a qualitative study. Ann Med. 2024;56:2401610. 10.1080/07853890.2024.2401610 39552336 PMC11574974

[33] Keller MS , Mosadeghi S, Cohen ER, Kwan J, Spiegel BMR. Reproductive health and medication concerns for patients with inflammatory bowel disease: thematic and quantitative analysis using social listening. J Med Internet Res. 2018;20:e206. 10.2196/jmir.9870 29891471 PMC6018236

[34] Zelinkova Z , Mensink PB, Dees J, Kuipers EJ, Van der Woude CJ. Reproductive wish represents an important factor influencing therapeutic strategy in inflammatory bowel diseases. Scand J Gastroenterol. 2010;45:46-50. 10.3109/00365520903362628 19883275

[35] Mountifield R , Andrews JM, Bampton P. It *is* worth the effort: Patient knowledge of reproductive aspects of inflammatory bowel disease improves dramatically after a single group education session. J Crohns Colitis. 2014;8:796-801. 10.1016/j.crohns.2013.12.019 24467964

[36] Maunder R , Toner B, De Rooy E, Moskovitz D. Influence of sex and disease on illness‐related concerns in inflammatory bowel disease. Can J Gastroenterol. 1999;13:728-732. 10.1155/1999/701645 10633825

[37] Walldorf J , Pijan E, Greinert R, Riesner-Wehner A, Michl P. Family planning with inflammatory bowel disease: the challenge of childlessness and parent concerns. Z Gastroenterol. 2021;59:841-850. 10.1055/a-1404-3610 33735917

[38] Druvefors E , Andersson RE, Hammar U, Landerholm K, Myrelid P. Minor impact on fertility in men with inflammatory bowel disease: a national cohort study from Sweden. Aliment Pharmacol Ther. 2022;56:292-300. 10.1111/apt.16984 35599362 PMC9322263

[39] Tavernier N , Fumery M, Peyrin-Biroulet L, Colombel JF, Gower-Rousseau C. Systematic review: fertility in non-surgically treated inflammatory bowel disease. Aliment Pharmacol Ther. 2013;38:847-853. 10.1111/apt.12478 24004045

[40] Banerjee A , Scarpa M, Pathak S, et al Inflammatory bowel disease therapies adversely affect fertility in men: a systematic review and meta-analysis. Endocr Metab Immune Disord Drug Targets. 2019;19:959-974. 10.2174/1871530319666190313112110 30864530

[41] Grosen A , Kelsen J, Lodberg Hvas C, Bellaguarda E, Hanauer SB. The influence of methotrexate treatment on male fertility and pregnancy outcome after paternal exposure. Inflamm Bowel Dis. 2017;23:561-569. 10.1097/MIB.0000000000001064 28267049

[42] Torres J , Chaparro M, Julsgaard M, et al European crohn’s and colitis guidelines on sexuality, fertility, pregnancy, and lactation. J Crohns Colitis. 2023;17:1-27. 10.1093/ecco-jcc/jjac115 36005814

[43] Friedman S , Magnussen B, OʼToole A, Fedder J, Larsen MD, Nørgård BM. Increased use of medications for erectile dysfunction in men with ulcerative colitis and Crohn’s disease compared to men without inflammatory bowel disease: a nationwide cohort study. Am J Gastroenterol. 2018;113:1355-1362. 10.1038/s41395-018-0177-6 29988041

[44] Pachler FR , Brandsborg SB, Laurberg S. Paradoxical impact of ileal pouch-anal anastomosis on male and female fertility in patients with ulcerative colitis. Dis Colon Rectum. 2017;60:603-607. 10.1097/DCR.0000000000000796 28481854

[45] Selinger CP , Eaden J, Selby W, et al Patients’ knowledge of pregnancy-related issues in inflammatory bowel disease and validation of a novel assessment tool (‘CCPKnow’). Aliment Pharmacol Ther. 2012;36:57-63. 10.1111/j.1365-2036.2012.05130.x https://##extlink46## 22568682

[46] Lai C , Sceats LA, Qiu W, Park KT, Morris AM, Kin C. Patient decision-making in severe inflammatory bowel disease: the need for improved communication of treatment options and preferences. Colorectal Dis. 2019;21:1406-1414. 10.1111/codi.14759 31295766

[47] Sanders R , Linn AJ. A mixed method study investigating the impact of talking about patients’ internet use on patient-reported outcomes. J Health Commun. 2018;23:815-823. 10.1080/10810730.2018.1514443 30351205

[48] Karimi N , Kanazaki R, Lukin A, Rotha Moore A, Williams AJ, Connor S. Clinical communication in inflammatory bowel disease: a systematic review of the study of clinician–patient dialogue to inform research and practice. BMJ Open. 2021;11:e051053. 10.1136/bmjopen-2021-051053 PMC8404434 34452967

[49] Dehara M , Wells MB, Sjöqvist H, Kosidou K, Dalman C, Sörberg Wallin A. Parenthood is associated with lower suicide risk: a register-based cohort study of 1.5 million swedes. Acta Psychiatr Scand. 2021;143:206-215. 10.1111/acps.13240 33011972 PMC7983926

[50] Elenkov A , Giwercman A, Søgaard Tøttenborg S, et al Male childlessness as independent predictor of risk of cardiovascular and all-cause mortality: a population-based cohort study with more than 30 years follow-up. PLoS One. 2020;15:e0237422. 10.1371/journal.pone.0237422 32881896 PMC7470262

