# Supplementary material for: Voluntary childlessness and family planning in men with inflammatory bowel disease: a scoping review
Source: Crohns Colitis 360. 2026 Jun 1;8(2):otag046. doi: 10.1093/crocol/otag046 (PMC13275309; doi:10.1093/crocol/otag046)
Supplement: otag046_Supplementary_Data [file otag046_supplementary_data.zip › Supplementary file 3.docx]

**Supplementary file 3: Original data extraction table**

Voluntary childlessness and family planning in men with inflammatory bowel disease: a scoping review

| Authors | Year | Study aim | Study design/data collection method | Country | Population | Total sample size (no. of men) | Key findings | Study limitations |
| --- | --- | --- | --- | --- | --- | --- | --- | --- |
| Maunder et al. | 1998 | To identify the concerns and sex differences of people with IBD. | Secondary analysis of cross-sectional survey data | Canada | IBD patients.  ≥18yrs.  All participants from a single tertiary care centre. | 343 (149) | Having children' was ranked of least concern in men, compared to being ranked 22/25 in women. Intimacy and sexual drive were ranked higher than 'having children' men. 'Energy levels' was the primary concern for both sexes. | Single centre.  Relationship status and ethnicity of study population not reported.  No control or comparison group.  Possible selection bias, 20% of participants undertook the survey when undergoing surgery.  Study not contemporary.  Findings may not be transferable. |
| Mountifield et al. | 2009 | To determine whether issues surrounding IBD, pregnancy and childbearing influence reproductive behaviour. | Cross-sectional postal survey | Australia | IBD patients.  18-59 yrs.  Recruited via a single tertiary care centre. | 217 (74) | Fear of infertility was significantly higher in female CD patients as compared to male CD patients (54% (n=47) female vs 32.5% (n=13) male). This gender-based difference was not reported in UC patients.  One male taking sulfasalazine attempting conception for 15 years before being informed of the reversible infertility associated with this agent. | Single centre. No control or comparison group. Socioeconomic and ethnic demographics of the participants not reported, possible selection and recall bias, findings may not be transferable.  Study not contemporary.  Most of the reported findings do not distinguish between male and female participants. |
| Sato et al. | 2009 | To investigate opinions among male IBD patients about pregnancy, conception and neonatal outcomes for partners. | Cross-sectional survey | Japan | IBD patients.  20-59yrs.  Recruited via a single tertiary care centre. | 364 (364) | 68% (n=154) of men who did not have children hoped to have a child in the future.  24.4% (n=71) of men who responded to the question reported hesitation about having children due to their disease. The rate of hesitation was significantly higher in CD than in UC patients (*p*=0.03). Significantly more men with CD than men with UC had undergone a therapy for sterility (*p*=0.03).  41.4% (n=151) considered that maintaining remission was needed at conception, and only 19.2% (n=70) considered a medication-free state was necessary. The safety of medication for inducing/maintaining remission was the most critical issue for male IBD patients when planning to conceive (51.4%, n=187). | Single centre. Socioeconomic and ethnic demographics of the participants not reported.  Possible recall bias, findings may not be transferable. |
| Zelinkova et al. | 2010 | To assess changes in medication in the peri-conceptional period. | Prospective cohort study | The Netherlands | IBD patients with active disease and desire to reproduce.  20-52years.  Recruited via a single outpatient clinic. | 61 ( 10) | On gastroenterologist advice, 5 men changed their medication due to plans for conception. Of these, 3 were advised to postpone or stop methotrexate, one was advised to stop an anti-TNF agent, and one was asked to stop treatment with 6-mercaptopurine. | Single centre.  Small sample size.  Socioeconomic and ethnic demographics of the participants not reported.  Possible selection bias in the recruitment methods, findings may not be transferable. |
| Mountifield, Andrews and Bampton | 2013 | To examine the effect of a single group education session on IBD-specific reproductive knowledge in subjects with IBD. | Non-randomised experimental study. | Australia | IBD patients (mean age 40.3yrs).  Advertised by a single tertiary centre. | 155 (48) | Baseline CCPKnow scores (knowledge of IBD specific aspects of reproduction) were significantly lower in males than females (4.8 vs 6.0). A significantly higher proportion of men demonstrated 'poor' knowledge at baseline than women (85.4% vs 56.4%). | Single centre.  Possible selection bias in study design.  Disease characteristics, socioeconomic and ethnic demographics of the participants not reported.  Findings may not be generalisable.  Data is not disaggregated by gender for a number of the study findings.  The CCPKnow score focuses on pregnancy and female issues. |
| Keller et al. | 2018 | To understand how individuals taking IBD medications during key reproductive periods make decisions about their medication use. | Qualitative research: social media content analysis | United States | IBD patients (age unknown) and their family and caregivers. | 1818 (male unknown) | 19 posts discussed male infertility or risks of birth defects due to medications taken by men with IBD. Several posts reported confusion or surprise that IBD medications could affect male fertility. One man with IBD was seeking advice online regarding taking methotrexate while trying to conceive. | The study was unable to confirm the diagnosis of those writing the post. Study unable to report demographic characteristics. Selection bias: data reflects only those engaged with and using social media and therefore findings may not be generalisable.  The study was not able to discern how many posts were made by men overall. |
| Rao et al. | 2020 | To assess counselling and knowledge about IBD and reproductive health. | Cross-sectional survey | United States | IBD patients.  18-45yrs. Recruited via a single tertiary care centre. | 100 ( 46) | 15% of men considered not having a child due to IBD. Reasons for considering voluntary childlessness in men were: risk of passing IBD to child, medication effects, inability to care for baby and self. 30% of men reported having being generally counselled on heritability, fertility or fertility and medication use. 62% of men wanted more information about IBD and reproductive health, mostly in the form of a handout of clinic visit. Median CCPKnow scores were low for men (3) and only 17% had adequate knowledge (a score of 8 or above). | Single centre. Possible recall bias of being counselled on reproductive issues. Study population had some selection bias to towards high-income patients. The small sample size and study design means that findings may not be generalisable. |
| Duricova et al. | 2021 | To investigate reproductive behaviour in patients with IBD in the Czech Republic. | Cross-sectional survey | Czech Republic | IBD patients. 18-65yrs.  Recruited from 22 centres. | 792 (272) | 47.1% of male participants reported already having offspring, 30.5% had at least one child unintentionally and 3.9% required reproductive assistance therapy. 69.4% were planning children in the future. 18.1% were voluntarily childless, of which 42.3% stated IBD was the cause of this decision.  22.4% of men reported fear of infertility. 11% of men reported impact of IBD on reproductive plans. Univariate analysis showed higher frequency of relapse, chronic active disease and ever use of biologics significantly increased changes to reproductive plans while ever use of biologics impacted fear of fertility. 20.2% had consulted a physician about reproductive issues, of which 76.4% were satisfied with the information. 29.4% were unsure of medication safety. | Author constructed survey (no validated survey available).  No control or comparison group.  Study population had more severe disease than the general IBD population.  Single geographical area. Socioeconomic and ethnic demographics of the participants not reported. |
| Fourie et al. | 2022 | To explore patient experiences of intimacy and sexuality in those living with IBD. | Qualitative research: participant interviews and anonymous narratives via online forms. | UK | People with IBD.  17-64yrs. | 43 (11) | Participants reported IBD affected family plans with some deciding on childlessness. One male participant reported the effect of medication on the possible child as an issue. | Disease characteristics, socioeconomic and ethnic demographics of the participants not reported.  Findings may not be generalisable due to sample size and study design. Focus of study is not parenthood so data is limited and only one male quote on the topic provided. |
| Winter et al. | 2022 | To assess the extent of and risk factors for voluntary childlessness in IBD. | Cross-sectional survey | United States | IBD patients.  ≥18 years.  Recruited via email through a specialist centre. | 464 (140) | 11 men (7.9% of all male participants) identified as being voluntarily childless compared to 18 women (5.6% of all female participants). 35 men had not yet thought about conceiving. Patients who were voluntarily childless were significantly younger than those who had or wanted children (*p*<0.0001), were diagnosed with IBD at an earlier age (*p*<0.0009), and had a longer disease duration (*p*<0.0069),. Additionally, patients who were voluntarily childless had significantly less formal education (*p*<0.019),. had significantly lower income ((*p*<0.0001),, and were more likely to be on permanent disability ((*p*<0.0001), than those who had or wanted children. IBD type and severity, including hospitalizations and surgeries, were not associated with increased frequency of VC. Of non-VC patients, 85 men planned for fewer children due to their IBD. The majority of patients in all groups responded that improved preconception counselling would not have influenced their decision to have children but would improve patients concerns regarding having children overall. Men and women who were voluntarily childless were significantly more concerned about finances as compared with those who had or wanted children (*p*<0.0094),. | Single centre. Possible selection bias as only recruited people with an email on file. No control or comparison group. Participants mostly white and female. Data is not disaggregated by gender for a number of the study findings. |
| Thapwong et al. | 2022 | To explore the lived experience of IBD patients and their family members regarding impacts of IBD on family members and their coping strategies. | Qualitative research: participant interviews | UK | People diagnosed with IBD for at least 1 year.  23-61yrs.  A 1st degree, co-habiting family member also interviewed.  Advertised via social media. | 6 participants with IBD (4)  6 participants that were partners of participants (2) | A female partner of a man with IBD reported concerns over the hereditary nature of Crohn's Disease. One man with IBD reported that "we stuck with one, and I think we wanted to give our daughter the best life we could, and I think perhaps we felt there would be too much pressure". | Findings may not be generalisable due to sample size and study design. Family planning and childlessness was not the focus of the study so data is limited on the topic. Not all findings in the study are disaggregated by gender. |
| Vieujean et al. | 2023 | To investigate healthcare professional and patient knowledge on fertility, pregnancy, and sexual function. | Cross-sectional online survey | Multiple European countries including France, Belgium, Italy and Poland | Healthcare professionals and adult IBD patients. 27-83yrs. | 793 patients (176) | 27.3% of men did not want children (compared to 14% of women), 35.2% did not have children but would like to have a child in the future, 13.1% already had a child or children and would like more.  30.5% of men reported an impact of IBD diagnosis on sexual function (vs 25% of women). 73.9% of men had not received any pre-conception counselling (vs 62% of women). 52% of men felt they had insufficient information regarding IBD and pregnancy (no difference in women). 75.4% of men felt comfortable discussing IBD and pregnancy with their IBD specialist (no difference in women). | No control population.  Possible selection bias (survey only available online and sent to patients who had an email within a healthcare registry). High level of missing data due to online collection method. No ethnicity data. No control or comparison group. No data provided on reasons for voluntary childlessness in men. |
| Erdmann et al. | 2024 | To understand how body experiences affect patients with IBD. | Qualitative research: participant interviews | Germany | IBD patients.  19-44yrs.  Recruited via outpatient clinics in two hospitals. | 10 (4) | One participant reported mixed feelings of fear and joy of having a third child. One participant reported voluntary childlessness due to a fear of own shortened life expectancy. | Disease characteristics, socioeconomic and ethnic demographics of the participants not reported.  Findings may not be generalisable due to sample size and study design. Focus of study not on parenthood/childlessness. |
| Gabova et al. | 2024 | To investigate the perceived impact of IBD on sexual life and family planning. | Qualitative research: participant interviews | Czech Republic | People with IBD.  21-67yrs.  Recruited through disease and patient organisations. | 36 (16) | The topic of family planning emerged in eight participants' interviews, only one of these was with a male participant. The study noted that male participants did not express concern for family planning in relation to IBD, though women did. For men in the study, topics frequently emerging were sexual activity, body image, discomfort and relationships. | Findings may not be generalisable due to sample size and study design. The study did not include many quotes from male participants, and no quote was included from the one male participant who had discussed family planning. More than half of the men in the study did not have any children, but the topic of voluntary childlessness was not discussed. Provides little insight into the impact of IBD on family planning from a male perspective. |
| Ma, Knapp and Galdas | 2024 | To describe and interpret the sexual health experiences of men with IBD. | Qualitative research: participant interviews | UK | IBD patients. 20-66yrs.  Recruited via outpatient clinics in three hospitals. | 22 (22) | Balancing the need to prioritise physical health with a desire for children was a difficult challenge for some men. The desire for children or having young children could affect treatment choices. Some men believed the disease would be a barrier to parenting, influencing decisions about when to have children. One participant expressed reluctance to progress to a proctectomy for fear of impotence, which he feared would influence his fertility. Another participant reported delaying stoma reversal due to concerns around destabilising his health while his son was young. One participant had considered child adoption prior to diagnosis but not revisited the idea following multiple surgeries. | Findings may not be generalisable due to sample size and study design. Ethnicity data not reported. |
