# Supplementary material for: Voluntary childlessness and family planning in men with inflammatory bowel disease: a scoping review
Source: Crohns Colitis 360. 2026 Jun 1;8(2):otag046. doi: 10.1093/crocol/otag046 (PMC13275309; doi:10.1093/crocol/otag046)
Supplement: otag046_Supplementary_Data [file otag046_supplementary_data.zip › Supplementary file 2.docx]

**Supplementary file 2: Search Strategy**

Voluntary childlessness and family planning in men with inflammatory bowel disease: a scoping review

**Database: OVID EMBASE 1974 to 2025 February 06**

**Search performed on the 7th February 2025**

1 exp ulcerative colitis/ or exp inflammatory bowel disease/ o exp Crohn Disease

2 crohn*.tw.

3 (ulcerativeadj1colitis).tw.

4 IBD.tw.

5 (inflammatoryadj1boweladj1disease*).tw..

6 1 or 2 or 3 or 5

7 exp male infertility/

8 exp reproductive behavior/ or exp reproductive health/

9 exp erectile dysfunction/

10 exp male sexual dysfunction/ or exp sexual behavior/ or exp sexual well-being/ or exp sexual function/ or exp sexual health/

11 exp men’s health/

12 childless*.tw.

13 (family adj1 plan*).tw.

14 father*.tw.

15 (parenting or parenthood).tw.

16 conception.tw.

17 preconception.tw.

18 contraceptive.tw

19 fertil*.tw

20 infertile*.tw.

21 7 or 8 or 9 or 10 or 11 or 12 or 13 or 14 or 15 or 16 or 17 or 18 or 19 or 20

22 men.mp. or male/

23 6 and 21 and 22

24 limited 23 to (human and English language)

**Database: OVID MEDLINE(R) ALL 1946 to February 06, 2025**

**Search performed on the 7th February 2025**

1 exp inflammatory bowel disease/ or exp colitis, ulcerative/ or exp crohn disease/

2 crohn*.mp.

3 (ulcerative adj1 colitis).mp

4 IBD.mp.

5 (inflammatory adj1 bowel adj1 diease*).mp.

6 1 or 2 or 3 or 4 or 5

7 exp infertility, Male/

8 exp Reproductive Rights/ or exp Reproductive Health Services/ or exp Reproductive Health/ or Reproductive Medicine/ or exp Reproductive Behavior or exp Reproductive History/

9 exp Contraception Behavior/ or exp Contraception

10 exp Erecile Dysfunction/

11 exp Sexual Behavior/ or exp Sexual Health/

12 exp Men’s Health/

13 childless*.mp.

14 family plan*.mp.

15 father*.mp.

16 parent*.mp.

17 conception.mp.

18 preconception.mp.

19 contraceptive.mp.

20 infertile*.mp.

21 fertil*.mp.

22 men.mp or exp Men/ or exp Male/

23 7 or 8 or 9 or 10 or 11 or 12 or 13 or 14 or 15 or 16 or 17 or 18 or 19 or 20 or 21

24 6 and 22 and 23

25 limit 24 to (English language and male and humans)

**Database: EBSCOHost CINAHL Complete**

**Search performed on the 7th February 2025**

S1 (MH “Inflammatory Bowel Diseases”)

S2 (MH “Crohn Disease”)

S3 (MH “Colitis, Ulcerative”)

S4 TI crohn* or AB crohn*

S5 TI “ulcerative colitis” or AB “ulcerative colitis”

S6 TI “inflammatory bowel disease” OR AB “inflammatory bowel disease”

S7 TI IBD or AB IBD

S8 S1 or S2 or S3 or S4 or S5 or S6 or S7

S9 (MH “Reproductive Behavior”) or (MH “Reproductive Health”) or (MHS “Ferility Nursing”)

S10 (MH “Contraceptive Agents, Male”)

S11 (MH “Contraception”)

S12 (MH “Sexual Health”) OR (MH “Sexual Counselling)

S13 (MH “Men’s Health”)

S14 TI childless” or AB childless*

S15 TI “family plan”” or AB “family plan*”

S16 TI father* or AB father*

S17 TI (parenting or parental or parenthood or parent-child or child-rearing or child rearing) or AB (parenting or parental or parenthood or parent-child or child-rearing or child rearing)

S18 TI (conception or preconception) or AB (conception or preconception)

S19 TI (contraception or both control or contraceptive*) or AB (contraception or both control or contraceptive*)

S20 TI infertile* or AB infertile*

S22 S9 or S10 or S11 or S12 or S13 or S14 or S15 or S16 or S17 or S18 or S19 or S20 or S21

S23 S8 AND S22

**APA PsychInfo 1806 to January 2025 Week 4**

**Performed on the 7th February 2025**

1 gastrointestinal disorders/ or colon disorders/

2 ulcerative colitis/

3 colon disorders/ or colitis/

4 crohn*.mp

5 colitis.mp.

6 IBD.mp.

7 (inflammatory adj1 bowel adj1 disease*).mp.

8 infertility/

9 infertility.mp

10 exp decision making/ or exp family planning attitudes/ or exp psychosexual behavior/ or exp reproductive health/ or contraceptive.mp. or exp family planning/ r exp birth control/ or exp coounseling/

11 Reproductive health/

12 childless*.mp.

14 father*.mp.

15 parent*.mp.

16 conception.mp.

17 preconception.mp.

18 contraceptive.mp.

19 infertile*.mp.

20 fertil*.mp.

21 8 or 9 or 10 or 11 or 12 or 13 or 14 or 15 or 16 or 17 or 18 or 19 or 20

22 1or 2 or 3 or 4 or 5 or 6 or 7

23 21 and 22

24 limit 23 to (human and English language)
